# Knockdown of NAA25 Suppresses Breast Cancer Progression by Regulating Apoptosis and Cell Cycle

**DOI:** 10.3389/fonc.2021.755267

**Published:** 2022-01-13

**Authors:** Jingkai Xu, Zhi Li, Xianbo Zuo, Guozheng Li, Xuejun Zhang, Bo Zhang, Yong Cui

**Affiliations:** ^1^ Department of Dermatology, China-Japan Friendship Hospital, Beijing, China; ^2^ Department of Dermatology, Jiangsu Province Hospital, Nanjing, China; ^3^ School of Life Sciences, Anhui Medical University, Hefei, China; ^4^ Department of Oncology, No. 2 Hospital, Anhui Medical University, Hefei, China; ^5^ Department of Dermatology, The First Affiliated Hospital of Anhui Medical University, Hefei, China

**Keywords:** breast cancer, NAA25, cell cycle, apoptosis, RNA sequencing

## Abstract

*NAA25* gene variants were reported as risk factors for type 1 diabetes, rheumatoid arthritis and acute arterial stroke. But it’s unknown whether it could contribute to breast cancer. We identified rs11066150 in *lncHSAT164*, which contributes to breast cancer, in our earlier genome-wide long non-coding RNA association study on Han Chinese women. However, rs11066150 A/G variant is also located in *NAA25* intron. Based on the public database, such as TCGA and Curtis dataset, *NAA25* gene is highly expressed in breast cancer tissues and this result has also been proved in our samples and cell lines through RT-qPCR and western blot analysis. To better understand the function of *NAA25* in breast cancer, we knocked down the expression of *NAA25* in breast cancer cell lines, FACS was used to detect cell apoptosis and cell cycle and colony formation assay was used to detect cell proliferation. We found that *NAA25*-deficient cells could increase cell apoptosis, delay G2/M phase cell and decrease cell clone formation. RNA sequencing was then applied to analyze the molecular profiles of *NAA25*−deficient cells, and compared to the control group, *NAA25* knockdown could activate apoptosis-related pathways, reduce the activation of tumor-associated signaling pathways and decrease immune response-associated pathways. Additionally, RT-qPCR was employed to validate these results. Taken together, our results revealed that NAA25 was highly expressed in breast cancer, and NAA25 knockdown might serve as a therapeutic target in breast cancer.

## Introduction

Breast cancer is the most common and a leading cause of cancer-related deaths of women worldwide (1). And China is undergoing the cancer transition stage, with the occurrence of female breast cancer increasing rapidly (2, 3). With the development of sequencing technologies, a lot of breast cancer associated genes have been validated (4, 5). Our previous case-control genome wide lncRNA association study on Han Chinese women identified that SNP rs11066150 was associated with breast cancer and *lncHSAT164* gene could contribute to breast cancer (6). And rs11066150 A/G was an intron variant in N-alpha-acetyltransferase 25 *(NAA25*) gene (also known as *MDM20*, *C12orf30* and *NAP1*). *NAA25* gene variants were reported to be associated with type 1 diabetes (T1D), rheumatoid arthritis, acute arterial stroke and dyslipidemia (7–10). However, the relationship between *NAA25* and breast cancer is still unknown.

*NAA25* encodes the auxiliary subunit, which could then affect posttranslational modifications by forming N-terminal acetyltransferase B complex with catalytic subunit *NAA20* (11). In yeast, it can regulate actin remodeling, and stabilize actin cytoskeleton and mitochondrial targeting (12, 13). And *NAA25* knockdown can disrupt cell cycle and reduce cell growth (14). However, the physiological function and mechanism of *NAA25* in breast cancer remain unknown.

To explore the relationship between *NAA25* gene and breast cancer, we compared *NAA25* gene expression between normal tissues and breast cancer tissues in public databases, such as TCGA and Curtis dataset, and analyzed the relationship between *NAA25* gene expression and overall survival (OS) of patients. In addition, we tested *NAA25* gene expression in breast cancer tissues, para-carcinoma tissues, breast cancer cell lines and normal breast epithelial cell lines. Furthermore, we specifically knocked down *NAA25* gene expression in breast cancer cells and explored its influence on tumor cell proliferation, apoptosis and cell cycle. Finally, RNA-seq analysis was used to clarify the molecular profiles of *NAA25*-deficient cells.

## Materials and Methods

### Subjects

In this study, four-pairs of breast cancer tissues and para-carcinoma tissues (all from Han Chinese women) were collected at the No.2 Hospital, Anhui Medical University. All cases were diagnosed with breast cancer by at least two pathologists. Para-carcinoma specimens were adipose/skin tissues, which were collected from breast cancer patients who underwent radical mastectomy. All tissue samples were stored in liquid nitrogen immediately after surgical resection. The information of breast cancer patients was provided in **Supplementary Table 1**.

### Cell Culture

MCF10A, MCF7, T47D, and HEK293T cell lines were purchased from the Institute of Basic Medical Sciences of the Chinese Academy of Medical Sciences. MCF10A, a kind of normal human breast epithelial cell, was grown in DMEM/F12 (Gibco, Life, China) medium supplemented with 10% fetal bovine serum (FBS) (Gibco, Australia), 10 µg/ml insulin (Macklin, China), 20 ng/ml EGF (Peprotech, China), and 0.5 µg/ml hydrocortisone (Macklin, China). MCF7 and HEK293T cells were maintained in DMEM (Gibco, Life, USA) supplemented with 10% FBS (Gibco, Australia). T47D cells were maintained in RPMI-1640 medium (Gibco, Life, USA) supplemented with 10% FBS (Gibco, Australia). All medium were supplemented with 100 U/ml penicillin–streptomycin (Gibco, Life, China), and all cells were maintained at 37°C in a humidified atmosphere containing 5% CO_2_ and confirmed to be mycoplasma free.

### RNA Extraction and RT-qPCR Analysis

The total RNA from the cell lines, human breast cancer tissues and para-cancerous tissues used in this study was extracted with TRIzol reagent, and DNase I (Thermo Fisher, USA) was used to remove genomic DNA. First-strand cDNA was synthesized by using the SuperScript III Reverse Transcriptase Kit (Thermo Fisher, USA). Relative RNA levels determined by RT-qPCR were measured on a Rotor-Gene Q real-time PCR machine (Qiagen, Germany). GAPDH was employed as an internal control. The relative expression of RNAs was calculated using the 2^−ΔΔCt^ method. All primer sequences for RT-qPCR are listed in **Supplementary Table 2**.

### Plasmid Construction, Transfection and Lentivirus Infection

Short hairpin RNAs (shRNAs) against NAA25 sh1 and sh2 were designed and synthesized by Taihe Biotechnology (Beijing, China) and cloned into the EGFP-Puro-pll3.7 plasmid. Based on the PSPAX2-PMD2G lentiviral system, a lentivirus was constructed according to the manufacturer’s instructions. After lentivirus infection, 1 µg/ml puromycin (*In vivo*Gen, USA) was added for selection, and 48-72 hours later, the cells were harvested for further experiments. shRNA sequences are listed in **Supplementary Table 2**.

### Western Blot Analysis

Tissues and cells were lysed in RIPA buffer (Beyotime, China). 40 μg of protein was used for SDS-PAGE gel electrophoresis (Bio-Rad) and transferred onto PVDF membranes (Millipore, China). Blocking was performed with 5% milk, and then the membranes were incubated with primary antibodies. Anti-NAA25 (1:1, 000 HPA039322, Sigma-Aldrich) or anti-actin (1:5000, A1978, Sigma-Aldrich) was added and incubated overnight at 4°C. After being washed, the membranes were incubated with secondary antibodies (peroxidase conjugated, suitable for each primary antibody) for 2 hours at room temperature. The signal was detected with a Bio-Rad ChemiDoc XRS + System after adding Super Signal West Pico chemiluminescence.

### Colony Formation Assay

To analyze cell growth, colony formation assays were performed. 1×10^3^ cells of T47D- and MCF7- Ctr, -sh1, -sh2 were seeded in a 6-well plate and incubated for 10 to 15 days at 37°C. Then, the cells were washed twice in PBS, fixed with 90% ethanol for 15 minutes and stained with 0.1% crystal violet for 20 minutes. Images of colonies were taken with a digital camera, and the number of colonies was analyzed by ImageJ v1.8.0 software.

### Apoptosis Assay

For apoptosis analysis, target cells were transferred to a 15 ml centrifuge tube, and annexin V binding buffer was added. After being centrifugated at 1,000 rpm for 5 min at 4°C, the cells were washed 3 times in PBS. Then, the cells were treated with 100 μl of binding buffer, 5 μl of Annexin V-APC and 1 μl of 100 μg/ml propidium iodide (PI) stain (Thermo Fisher, USA), and incubated in the dark for 25 min. Cell apoptosis was analyzed by flow cytometry (BD Biosciences).

### Cell Cycle Assay

For cell cycle analysis, target cells were fixed with 75% ice-cold ethanol at 4°C overnight. Then, the cells were suspended in PBS supplemented with 100 mg/ml RNase A for 30 min at 37°C and then stained with 50 µg/ml PI (Thermo Fisher, USA) in the dark at room temperature for 15 min. Finally, a total of 20,000 cells were analyzed on a FACS Calibur flow cytometer equipped with Cell Quest software (BD Biosciences).

### RNA-Seq Analysis

After *NAA25* knockdown in T47D cells, cells from the Ctr, sh1 and sh2 groups were harvested for RNA-seq analysis at Shanghai Majorbio Biopharm Technology Co. mRNAs were isolated from total RNA with the oligo (dT) method. The mRNAs were fragmented, and then first-strand cDNA and second-strand cDNA were synthesized. After being purified, cDNA fragments were linked to adapters. Then, cDNA fragments of suitable size were selected for PCR amplification. The sequencing platform used in this study was Illumina HiSeq, and the paired-end reads were 2×150 bp. TPM (Transcripts Per Million reads) was used to evaluate genes expression, transcript abundance was assessed with the DESeq2, and the significantly affected genes were determined by setting a fold change of ≥ 2. The differentially expressed gene (DEG), Kyoto Encyclopedia of Genes and Genomes (KEGG) pathway, gene ontology (GO), GO term and gene set enrichment analysis (GSEA) described in this paper were performed on the free online platform Majorbio Cloud Platform (www.majorbio.com).

### Statistical Analysis

All statistical analyses were performed using Graphpad Prism 8.0 statistical software (California, US). Experiment data are shown as the means ± SEM, and all experiments were conducted for at least three times. Significance was determined using the Student’s *t*-test: N.S. *p* > 0.05; ∗*p* < 0.05; ∗∗*p* < 0.01; ∗∗∗*p* < 0.001 and ∗∗∗∗*p* < 0.0001.

## Results

### rs11066150 Associated Gene NAA25 Highly Expressed in Breast Cancer

**Figure 1:**
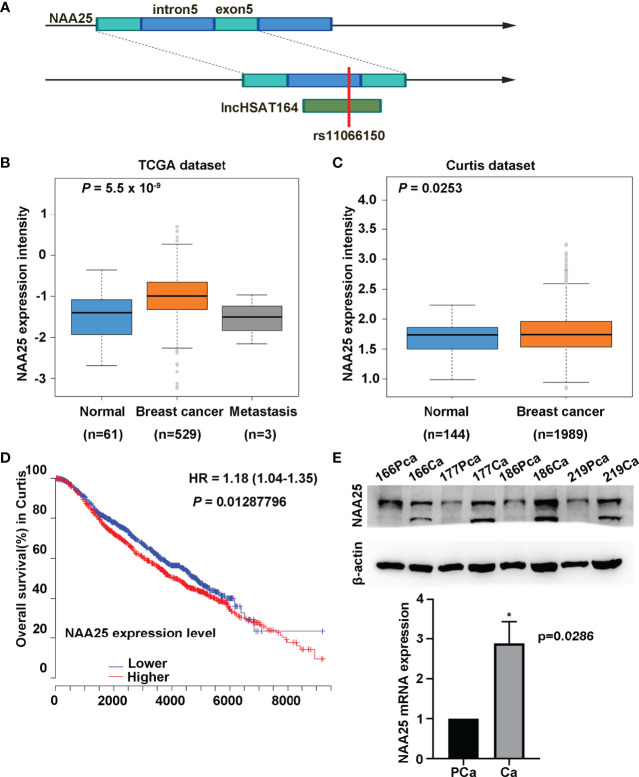
Characterization of NAA25 gene in breast cancer. **(A)** rs11066150 variant schematic diagram in lncHSAT164 and NAA25. **(B, C)** NAA25 gene was highly expressed in breast cancer tissues compared to the controls in TCGA dataset and Curtis dataset. **(D)** OS analysis of patients with high and low NAA25 expression. The *p* value was calculated using Mann-Whitney *U* tests. **(E)** Western-blot and RT-qPCR to analyze NAA25 expression in breast cancer tissues and para-carcinoma tissues, NAA25 was highly expressed in breast cancer tissues. The two bands are all *NAA25*. *p < 0.05.

rs11066150 was reported in *lncHSAT164* (6), and it is also located within the fifth intron of *NAA25* gene (**Figure 1A**). Based on the eQTLGen database (https://www.eqtlgen.org/), we identified 4 cis-eQTL effects genes, *TMEM116*, *HECTD4*, *MAPKAPK5* and *NAA25*, to be associated with rs11066150 (**Supplementary Table 3**). And *TMEM116*, *HECTD4*, *MAPKAPK5* was reported to be associated with renal cell carcinoma, prostate cancer and colorectal cancer (15–17). However, *NAA25* gene has never been reported to be associated with cancers.

**Figure 2:**
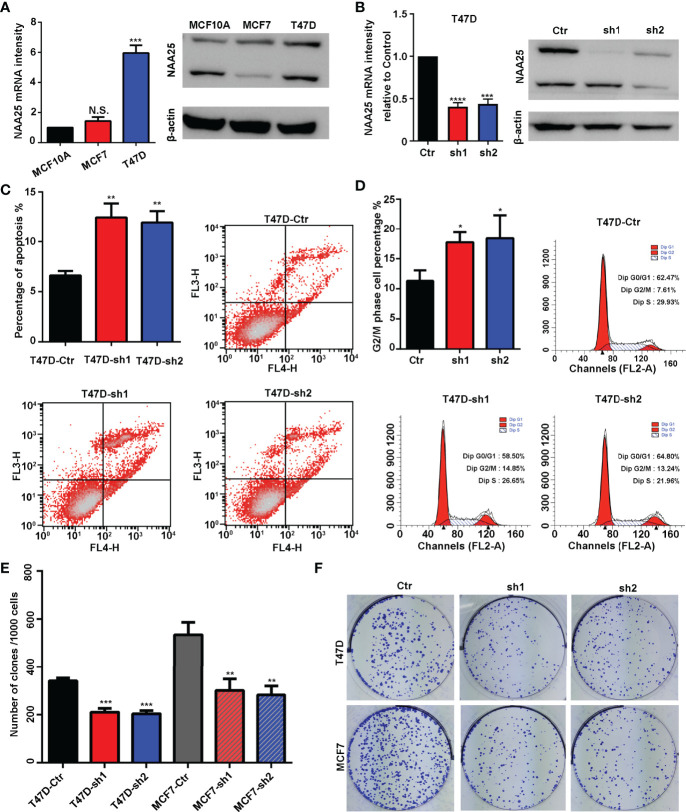
NAA25 gene influences cell apoptosis and the cell cycle in breast cancer. **(A)** RT-qPCR and western blot analysis of NAA25 gene expression in breast cancer cell lines (The two bands are all *NAA25*.). **(B)** RT-qPCR and western blot analysis in the NAA25-deficient T47D cell line. **(C)** Cell apoptosis in the NAA25-deficient T47D cells. Compared to the Ctr group, NAA25 knockdown could increase cell apoptosis. **(D)** Cell cycle analysis of the NAA25-deficient T47D cells. Compared to the Ctr group, NAA25 knockdown induced G2/M cell cycle arrest. **(E, F)** Downregulated NAA25 reduced the clonogenic potential of breast cancer cells. N.S. *p* > 0.05; *p < 0.05; **p < 0.01; ***p < 0.001 and ****p < 0.0001.

To explore the role of *NAA25* gene in breast cancer, we analyzed its expression in different public databases. According to TCGA and the Curtis, Finak breast and Richardson breast datasets (18–21), we found that *NAA25* was greatly up regulated in breast cancer tissues in comparison with normal breast tissues (**Figures 1B, C** and **Supplementary Figures 1A, B**). Furthermore, high mRNA levels of *NAA25* showed marginal associations with poor OS in the Curtis database (*p* = 0.013) (**Figure 1D**). Additionally, we explored the expression of *NAA25* gene in breast cancer tissues and para-cancerous tissues. RT-qPCR and western blot analyses were performed in four-pairs of tissues, and results revealed that *NAA25* was highly expressed in cancer tissues (**Figure 1E**). We also monitored *NAA25* expression in normal breast epithelial cell line MCF10A, and breast cancer cell lines MCF7 and T47D. Compared to MCF10A, *NAA25* was highly expressed in T47D cells (**Figure 2A**). Together, our analyses reveal a previously unknown role of *NAA25* in breast cancer, and highly expressed *NAA25* might influence the progress of breast cancer.

### NAA25 Knockdown Inducing Apoptosis, G2/M Arrest and Suppressing Cell Proliferation

To investigate the physiological roles of *NAA25* gene in breast cancer, two shRNA targets were designed to knockdown *NAA25* gene in breast cancer cell lines, and the mRNA expression and protein expression of *NAA25* were both significantly diminished (**Figure 2B** and **Supplementary Figure 1C**). Apoptosis is a key cellular process in breast cancer. We measured the effect of *NAA25* on apoptosis and cell cycle by FACE analysis. Compared to the Ctr group, the number of apoptotic cells was relatively larger in the shRNA groups (*p* < 0.01), as shown in (**Figure 2C**), and more cells were arrested in the G2/M phase (*p* < 0.05), as shown in (**Figure 2D**).

To further investigate whether NAA25 knockdown could influence tumor growth, colony formation assays were applied in this study, which illustrated that clonogenic survival significantly decreased following *NAA25* knockdown in T47D cell line (**Figures 2E, F**). And similar results were investigated in NAA25-deficient MCF7 cells. Hence, based on these results, we conclude that *NAA25* is highly expressed in breast cancer and may lead to poor OS in patients by regulating tumor cell apoptosis and cell cycle.

### RNA Sequencing Characterizing the Molecular Profile of NAA25-Deficient Breast Cancer Cells

**Figure 3:**
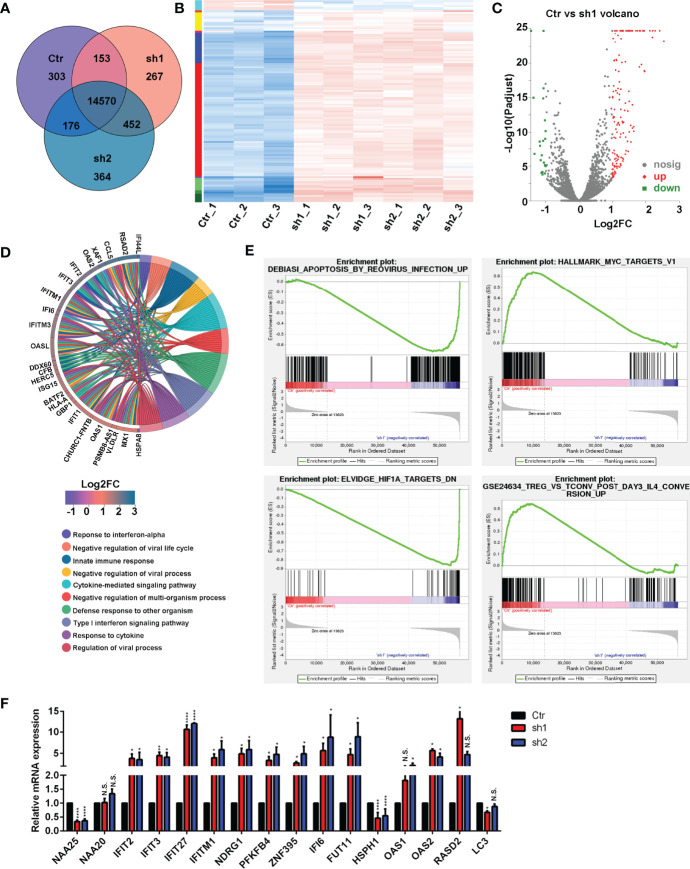
RNA-seq analysis in the NAA25-deficient T47D cells. **(A)** Venn diagram analysis of gene or transcript expression among the Ctr group and shRNA groups. **(B)** Differentially expressed gene (DEG) heatmap analysis. Blue indicates downregulated genes. Red indicates upregulated genes. **(C)** Volcano plot showing the DEG in the Ctr group and the sh1 group. **(D)** GO term analysis between the Ctr group and the sh1 group. **(E)** Gene set enrichment analysis (GSEA) to analyze DEG between the Ctr group and the sh1 group. **(F)** RT-qPCR analysis to validate DEGs after NAA25 knockdown in the T47D cells. The data shown here are representative of at least 3 independent experiments. N.S. *p* > 0.05; *p < 0.05; **p < 0.01 and ****p < 0.0001.

To investigate the importance of *NAA25* gene in breast cancer, RNA-seq analysis was applied after *NAA25* knockdown in the T47D cell line. Pearson’s correlation analysis (PCA) was performed to cluster all samples (**Supplementary Figure 2A**). Based on the gene expression matrix, the Venn diagram was used to analyze the co-expressed and specifically expressed genes or transcripts among the Ctr group and shRNA groups (**Figure 3A**). Furthermore, differentially expressed gene (DEG) analysis was conducted to compare the Ctr group and the sh1 and sh2 groups respectively, and 119 DEGs were identified (**Figures 3B, C**, **Supplementary Figure 2B**). All DEGs were presented in **Supplementary Table 4**.

Furthermore, KEGG enrichment analyses were performed among the 119 DEGs, and most of them were related to infections, immune responses, cancers and immune diseases (**Supplementary Figure 2C**). GO term analysis was performed to *NAA25*-deficient cells, and the results showed that many genes were associated with infection and immunity (**Figure 3D** and **Supplementary Figure 2D**).

To assess the molecular pathways involved in *NAA25*-deficient T47D cells, we performed gene set enrichment analysis (GSEA). And *NAA25* knockdown could increase apoptosis associated pathways, and reduce tumor associated pathways, like MYC, HIF1A, ERB2, MEK and TNF (**Figure 3E** and **Supplementary Figure 2E**). In addition, immune response associated pathways like IL4, TNF and LTE2 were reduced. Finally, RT-qPCR analysis was used to verify RNA-seq data (**Figure 3F**). *IFIT2*, *IFIT3*, *IFIT27*, *IFITM1*, *NDRG1*, *PFKFB4*, ZN*F395*, *IFI6*, *FUT11* and *OAS2* mRNA expression was upregulated after *NAA25* knockdown, and *HSPH1* gene expression was down regulated, consistent with the RNA-seq results.

## Discussion

A large number of breast cancer associated susceptibility SNPs and genes were identified and reported as a molecular marker in tumor incidence, metastasis, prognosis and treatment. Previously, we performed a genome-wide lncRNA association study in Han Chinese women and identified two new susceptibility SNPs, rs11066150 and rs12537 (6) (22). rs11066150 variant had no relationship with the clinical characteristics of breast cancer like family history, menopausal status, and molecular subtypes (22). However, rs11066150 associated lncRNA, *lncHSAT164*, was highly expressed in breast cancer, and overexpressed *lncHSAT164* could promote colony formation and down-expressed *lncHSAT164* could promote cell apoptosis and regulate cell cycle (6). In this study, we reported rs11066150 as an intron variant SNP in *NAA25* gene. And *NAA25* gene is highly expressed in breast cancer tissues relative to normal tissues, while high *NAA25* expression is correlated with poor OS. And *NAA25* knockdown could induce cell apoptosis, delay G2/M phase cell and decrease cell clone formation. *NAA25* was reported to be associated with T1D (7, 23), arthritis (8, 24) and virus infection (25). However, *NAA25* gene was reported as a proto-oncogene in breast cancer for the first time, and more research is needed in the future to characterize the impact of rs11066150 A/G variant on breast cancer, and the relationship between *lncHSAT164* and *NAA25* gene also needs further study.

RNA-seq is a ubiquitous tool in molecular biology that is shaping nearly every aspect of our understanding of genomic function (26). The molecular features of *NAA25*-deficient T47D cell lines were analyzed by RNA-seq in this work, and analysis results indicated that many infection and immune associated genes were highly expressed, which suggests that immune therapy may be an effective approach in treating *NAA25*-overexpressed breast cancer.

Highly expressed *IFIT2* and *NDRG1* could reduce tumor migration and metastasis (27–30). And *HSPH1* was highly expressed in different tumors, such as colorectal cancer, B-cell lymphoma, melanoma and esophageal squamous cell carcinoma (31–34), while *NAA25* knockdown could upregulate *IFIT2* and *NDRG1* expression and downregulate *HSPH1* expression (**Figure 3F**). These findings suggest that *NAA25* knockdown may also play a positive role in treating other cancers. As an important accessory subunit of the NatB enzymatic complex, *NAA25* could work with the *NAA20* catalytic subunit to promote enzymatic activity (26, 35), and *NAA25* knockdown did not reduce *NAA20* expression (12). It’s also verified in the current study.

In conclusion, in this study, we reported *NAA25* as a candidate gene of rs11066150, which was highly expressed in breast cancer, and highly expressed *NAA25* could reduce patient’s OS. In addition, *NAA25* knockdown could induce cell apoptosis, delay G2/M phase cell and decrease cell clone formation. RNA-seq analysis was also applied to clarify the molecular profiling of *NAA25*-deficient cells, and *NAA25* knockdown repressed tumor- and immune response-associated pathways. This study is among the first attempts to clarify the function of *NAA25* in breast cancer, and these results have elucidated the mechanism of *NAA25* in breast cancer and suggests that *NAA25* may serve as a potential therapeutic target of breast cancer.

## Data Availability Statement

The datasets presented in this study can be found in online repositories. RNA-seq data presented in the study are deposited in the SRA repository, accession number PRJNA752396. Further inquiries can be directed to the corresponding authors.

## Ethics Statement

This study was approved by the Ethics Committee of Anhui Medical University. The patients/participants provided their written informed consent to participate in this study.

## Author Contributions

JX, YC, and BZ conceived of the idea. JX, ZL, and GL performed the experiments. JX, XBZ, and XJZ analyzed the data. JX drafted the manuscript. All authors contributed to the article and approved the submitted version.

## Funding

This work was supported by the scientific and technological innovation leading talents of “Ten Thousand Talents Program” (2018-WRJH-1), the National Natural Science Foundation of China (81872516), the discipline construction project of Peking Union Medical College (xhxk201903) and 2020 medical service and support capability upgrade project (2020-QTL-008).

## Conflict of Interest

The authors declare that the research was conducted in the absence of any commercial or financial relationships that could be construed as a potential conflict of interest.

The reviewer, H-FZ, declared a past co-authorship with one of the authors, XJZ, to the handling editor.

## Publisher’s Note

All claims expressed in this article are solely those of the authors and do not necessarily represent those of their affiliated organizations, or those of the publisher, the editors and the reviewers. Any product that may be evaluated in this article, or claim that may be made by its manufacturer, is not guaranteed or endorsed by the publisher.

## Supplementary Material

Supplementary Figure 1 NAA25 gene expression in public databases. **(A, B)** Compared to normal tissues NAA25 gene was highly expressed in breast cancer tissues in Finak breast and Richardson breast databases. **(C)** RT-qPCR and western blot analysis in the NAA25-deficient MCF7 cell line. The two bands are all *NAA25*. *p* < 0.05; ***p* < 0.001.

Supplementary Figure 2 RNA-seq analysis in the NAA25-deficient T47D cells. **(A)** Pearson’s correlation analysis (PCA) clarified the similarity between RNA-seq samples. **(B)** Volcano plot showing the DEG in the Ctr group and the sh2 group. **(C)** GO term analysis between the Ctr group and the sh1 group. **(D)** GO analysis between the Ctr group and the sh1 group. **(E)** Gene set enrichment analysis (GSEA) to analyze the DEG between the Ctr group and the sh2 group.
