# Supplementary figures and images for: Knockdown of NAA25 Suppresses Breast Cancer Progression by Regulating Apoptosis and Cell Cycle
Source: Front Oncol. 2022 Jan 13;11:755267. doi: 10.3389/fonc.2021.755267 (PMC8792228; doi:10.3389/fonc.2021.755267)

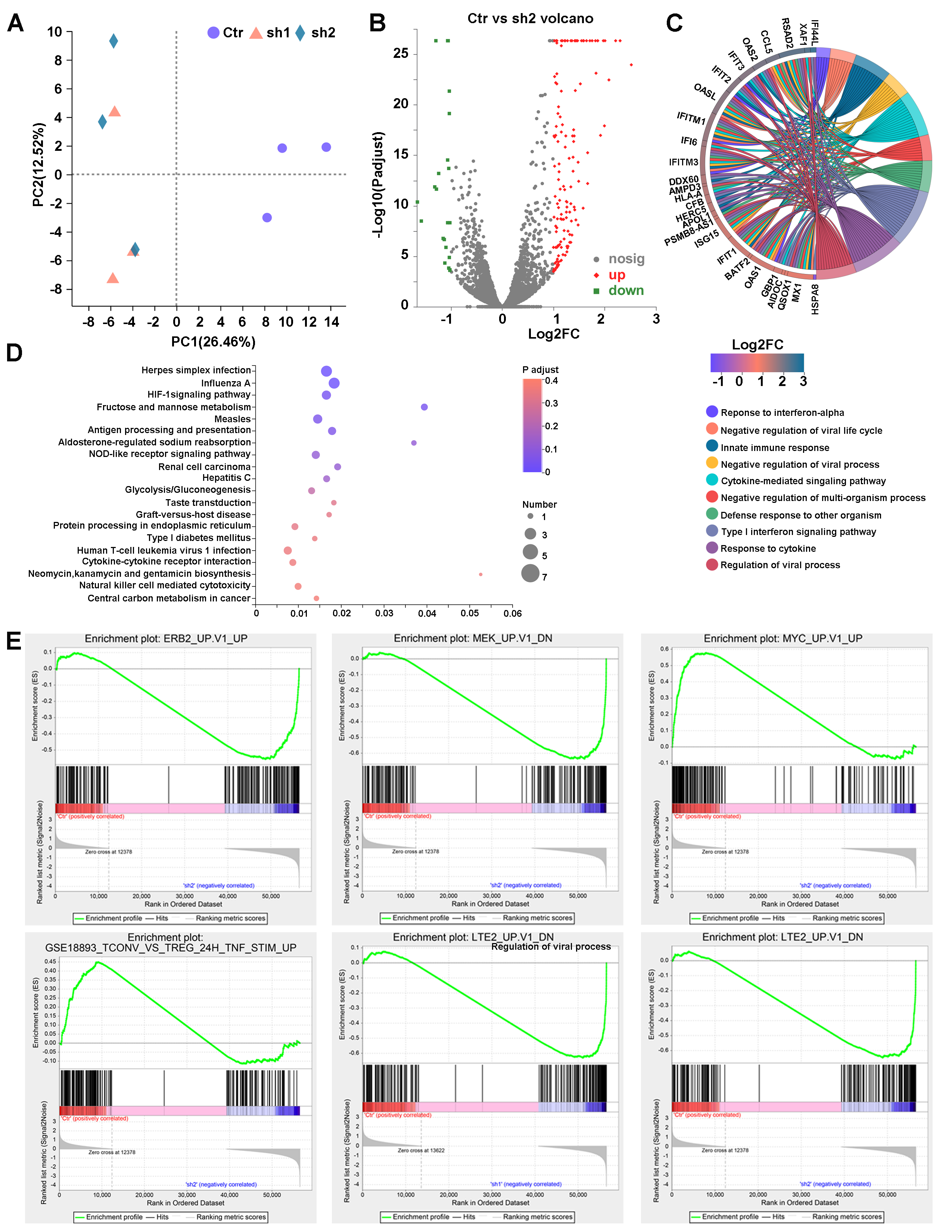

Supplement: Supplementary Figure 1 — NAA25 gene expression in public databases. (A, B) Compared to normal tissues NAA25 gene was highly expressed in breast cancer tissues in Finak breast and Richardson breast databases. (C) RT-qPCR and western blot analysis in the NAA25-deficient MCF7 cell line. The two bands are all NAA25. p < 0.05; **p < 0.001. [file Image_1.tif]

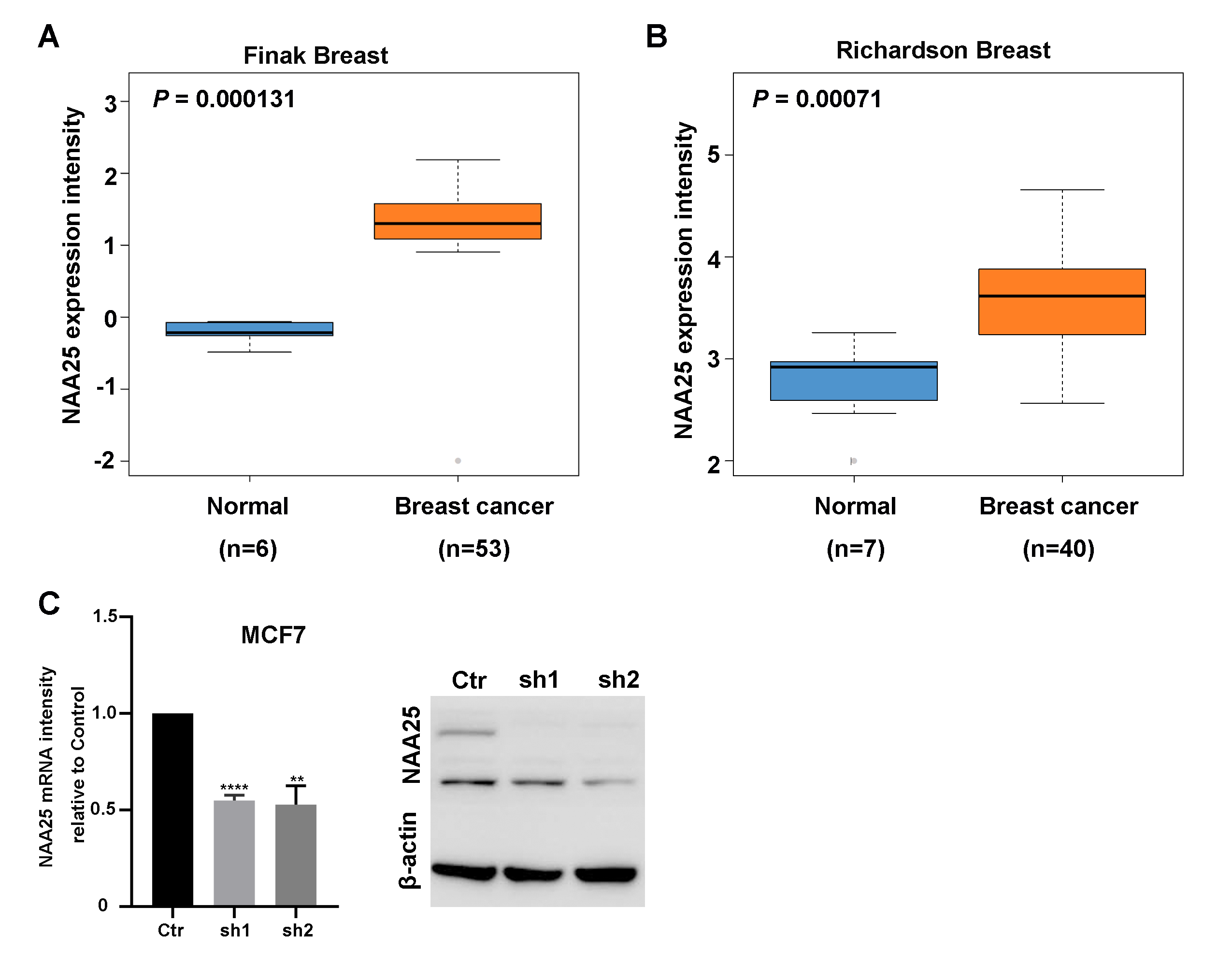

Supplement: Supplementary Figure 2 — RNA-seq analysis in the NAA25-deficient T47D cells. (A) Pearson’s correlation analysis (PCA) clarified the similarity between RNA-seq samples. (B) Volcano plot showing the DEG in the Ctr group and the sh2 group. (C) GO term analysis between the Ctr group and the sh1 group. (D) GO analysis between the Ctr group and the sh1 group. (E) Gene set enrichment analysis (GSEA) to analyze the DEG between the Ctr group and the sh2 group. [file Image_2.tif]
