# Supplementary material for: Knockdown of NAA25 Suppresses Breast Cancer Progression by Regulating Apoptosis and Cell Cycle
Source: Front Oncol. 2022 Jan 13;11:755267. doi: 10.3389/fonc.2021.755267 (PMC8792228; doi:10.3389/fonc.2021.755267)
Supplement: Supplementary file 3 [file DataSheet_1.docx]

Supplementary Material

# Supplementary Figures and Tables

## Supplementary Figures

**Supplementary Figure 1.** NAA25 gene expression in public databases. **(A-B)** Compared to normal tissues NAA25 gene was highly expressed in breast cancer tissues in Finak breast and Richardson breast databases. **(C)** RT-qPCR and western blot analysis in the NAA25-deficient MCF7 cell line. *p* < 0.05; **, *p* < 0.001.

**Supplementary Figure 2.** RNA-seq analysis in the NAA25-deficient T47D cells. **(A)** Pearson’s correlation analysis (PCA) clarified the similarity between RNA-seq samples. **(B)** Volcano plot showing the DEG in the Ctr group and the sh2 group. **(C)** GO term analysis between the Ctr group and the sh1 group. **(D)** GO analysis between the Ctr group and the sh1 group. **(E)** Gene set enrichment analysis (GSEA) to analyze the DEG between the Ctr group and the sh2 group.

## Supplementary Tables

**Supplementary Table1 Characteristics of breast cancer patients**

| **Sample Number** | **Sex** | **Age** | **Staging** | **ER**  **Positive** | **PR**  **Positive** | **HER2**  **Positive** | **Ki67**  **(%)** | **Molecular**  **Subtypes** | **Opreation** | **Chemotherapy** |
| --- | --- | --- | --- | --- | --- | --- | --- | --- | --- | --- |
| TBC166 | Female | 44 | Ⅱ | Yes | Yes | No | 10 | Lumina A breast cancer | Modified radical mastectomy | No |
| TBC177 | Female | 66 | Ⅱ | Yes | Yes | No | 8 | Lumina A  breast cancer | Modified radical mastectomy | No |
| TBC186 | Female | 64 | Ⅱ | No | No | Yes | 10 | HER-2 Amplified | Modified radical mastectomy | No |
| TBC219 | Female | 71 | Ⅱ | Yes | Yes | No | 8 | Lumina A  breast cancer | Modified radical mastectomy | No |

**Supplementary Table2 Primer and shRNA sequences**

| **Gene Name** | **Primers Name** | **Primer Sequences (5’-3’)** |
| --- | --- | --- |
| NAA25 | NA5-F | TGGAGCTGATTAGGCGTTTAC |
|  | NA5-R | TTCTCTGACCACACTCAATTTCT |
| GAPDH | q GAPDH-F | GTGAAGGTCGGAGTCAACG |
|  | q GAPDH-R | TGAGGTCAATGAAGGGGTC |
| NAA25-sh1 | shRNA1-F | TCGAGGCTGTGAAGTTTATAGAAGATTTCAA  GAGAATCTTCTATAAACTTCACAGCTTTTTGC |
|  | shRNA1-R | GGCCGCAAAAAGCTGTGAAGTTTATAGAAGA  TTCTCTTGAAATCTTCTATAAACTTCACAGCC |
| NAA25-sh2 | shRNA2-F | TCGAGGCAGATAAACTGTTGAAGAAATTCAA  GAGATTTCTTCAACAGTTTATCTGCTTTTTGC |
|  | shRNA2-R | GGCCGCAAAAAGCAGATAAACTGTTGAAGA  AATCTCTTGAATTTCTTCAACAGTTTATCTGCC |
| NAA20 | NAA20-4F | GGGCTACAGTGTATATAGGACGG |
|  | NAA20-4R | ATCCCTGGAAAGTGCTTTCCTC |
| IFIT2 | IFIT2-2F | CACGCTGTGGCTCATCTGAAG |
|  | IFIT2-2R | ATCTGCTAGAGCATGGAGGCT |
| IFIT3 | IFIT3-F | AGAAAAGGTGACCTAGACAAAGC |
|  | IFIT3-R | CCTTGTAGCAGCACCCAATCT |
| IFI27 | IFI27-1F | TGCTCTCACCTCATCAGCAGT |
|  | IFI27-1R | CACAACTCCTCCAATCACAACT |
| IFITM1 | IFITM1-1F | CCAAGGTCCACCGTGATTAAC |
|  | IFITM1-1R | ACCAGTTCAAGAAGAGGGTGTT |
| NDRG1 | NDRG1-1F | CTCCTGCAAGAGTTTGATGTCC |
|  | NDRG1-1R | TCATGCCGATGTCATGGTAGG |
| PFKFB4 | PFKFB4-2F | GCTATGAGAACTCCTACGAGTCGC |
|  | PFKFB4-2R | CACGTAGCTCTGGCCCACAT |
| ZNF395 | ZNF395-2F | GGGCATCAAACGACACGTCAA |
|  | ZNF395-2R | CCTTCAGCTGCACCTCTGTG |
| IFI6 | IFI6-2F | AGTGGCCACGCTGCAGAG |
|  | IFI6-2R | CTCCTCATCCTCCTCACTATCG |
| FUT11 | FUT11-2F | ACCTGGCTTTGGCAATGTGGA |
|  | FUT11-2R | TTCCCCCTGGTCCAGACCTT |
| HSPH1 | HSPH1-1F | ATGAGTTCAGCGACCGGTG |
|  | HSPH1-1R | GGCTGCAACTCCGATTGTTCT |
| RSAD2 | RSAD2-2F | CACTAAACCCTGTCCGCTGGA |
|  | RSAD2-2R | GTGGCGCTCCAAGAATCTTTCA |
| OAS1 | OAS1-1F | TGCCTGCCTTTGATGCCCTG |
|  | OAS1-1R | CTCTTTCTGCAGGTCGGTGCA |
| OAS2 | OAS2-1F | CTGCACCAGCTCCAATCAGC |
|  | OAS2-1R | AGGTTGGGAGAAGTCAACCAGG |
| LC3 | LC3-F | ACTCCTGACTGCATGGAAGC |
|  | LC3-R | GTCCACAGCTGCTTTTCCAC |

**Supplementary Table 3 rs11066150 associated cis-eQTL effects genes**

| ***p* value** | **Gene ID** | **Gene Symbol** | **Chr** | **Pos (hg19)** | **Z-score** | **FDR** |
| --- | --- | --- | --- | --- | --- | --- |
| 2.38E-29 | ENSG00000198270 | TMEM116 | 12 | 112410027 | 11.2477 | 0 |
| 4.02E-27 | ENSG00000173064 | HECTD4 | 12 | 112708944 | 10.7859 | 0 |
| 9.40E-13 | ENSG00000089022 | MAPKAPK5 | 12 | 112307062 | -7.139 | 0 |
| 5.76E-09 | ENSG00000111300 | NAA25 | 12 | 112505663 | -5.8235 | 3.88149E-05 |

**Supplementary Table4 DEGs among Ctr group, sh1 group and sh2 group**

| **Gene ID** | **Gene Name** | **Ctr_1** | **Ctr_2** | **Ctr_3** | **sh1_1** | **sh1_2** | **sh1_3** | **sh2_1** | **sh2_2** | **sh2_3** |
| --- | --- | --- | --- | --- | --- | --- | --- | --- | --- | --- |
| ENSG00000111335 | OAS2 | -0.3723 | -0.3855 | -0.4246 | 0.1620 | 0.2279 | 0.1504 | 0.2381 | 0.1836 | 0.2203 |
| ENSG00000126709 | IFI6 | -0.3719 | -0.3116 | -0.4146 | 0.2302 | 0.1121 | 0.2470 | 0.1875 | 0.1623 | 0.1592 |
| ENSG00000142089 | IFITM3 | -0.3276 | -0.3208 | -0.4194 | 0.2169 | 0.1783 | 0.1861 | 0.1315 | 0.1627 | 0.1922 |
| ENSG00000137965 | IFI44 | -0.3421 | -0.3685 | -0.3649 | 0.2125 | 0.2245 | 0.1123 | 0.1686 | 0.0993 | 0.2583 |
| ENSG00000185885 | IFITM1 | -0.3344 | -0.3481 | -0.5013 | 0.2501 | 0.2333 | 0.1603 | 0.1532 | 0.1749 | 0.2121 |
| ENSG00000108679 | LGALS3BP | -0.3096 | -0.2690 | -0.3638 | 0.1049 | 0.0873 | 0.2247 | 0.2247 | 0.1800 | 0.1210 |
| ENSG00000206503 | HLA-A | -0.3022 | -0.2670 | -0.3815 | 0.1243 | 0.0984 | 0.2477 | 0.1820 | 0.2234 | 0.0748 |
| ENSG00000143365 | RORC | -0.2935 | -0.2854 | -0.3722 | 0.1456 | 0.1555 | 0.1568 | 0.2278 | 0.2271 | 0.0383 |
| ENSG00000186918 | ZNF395 | -0.2876 | -0.3077 | -0.4742 | 0.1490 | 0.1656 | 0.1731 | 0.2139 | 0.2421 | 0.1259 |
| ENSG00000258102 | MAP1LC3B2 | -0.3265 | -0.2410 | -0.3987 | 0.1152 | 0.1212 | 0.1944 | 0.1282 | 0.2578 | 0.1495 |
| ENSG00000196968 | FUT11 | -0.2888 | -0.2193 | -0.4034 | 0.1319 | 0.1591 | 0.1426 | 0.1928 | 0.1958 | 0.0893 |
| ENSG00000258667 | HIF1A-AS2 | -0.2440 | -0.3465 | -0.4011 | 0.1601 | 0.2033 | 0.0441 | 0.2153 | 0.1771 | 0.1917 |
| ENSG00000206337 | HCP5 | -0.2617 | -0.2386 | -0.4175 | 0.1051 | 0.1001 | 0.2113 | 0.1836 | 0.2258 | 0.0917 |
| ENSG00000168447 | SCNN1B | -0.2522 | -0.2941 | -0.4871 | 0.2055 | 0.1492 | 0.1954 | 0.2211 | 0.1964 | 0.0657 |
| ENSG00000257607 | AC073957.2 | -0.4220 | -0.3412 | -0.3432 | 0.1486 | 0.2520 | 0.1181 | 0.3137 | 0.2364 | 0.0377 |
| ENSG00000230873 | STMND1 | -0.2409 | -0.2322 | -0.4393 | 0.1650 | 0.1166 | 0.1564 | 0.1808 | 0.2111 | 0.0825 |
| ENSG00000129451 | KLK10 | -0.2953 | -0.1804 | -0.3999 | 0.0922 | 0.1259 | 0.2097 | 0.1909 | 0.2019 | 0.0548 |
| ENSG00000167772 | ANGPTL4 | -0.3476 | -0.2630 | -0.5463 | 0.2311 | 0.1319 | 0.2022 | 0.2744 | 0.2374 | 0.0798 |
| ENSG00000132530 | XAF1 | -0.5077 | -0.3916 | -0.3958 | 0.1535 | 0.2930 | 0.1865 | 0.2370 | 0.2094 | 0.2157 |
| ENSG00000119917 | IFIT3 | -0.4032 | -0.4356 | -0.3562 | 0.2774 | 0.2534 | 0.0921 | 0.2030 | 0.1357 | 0.2333 |
| ENSG00000119922 | IFIT2 | -0.4182 | -0.4322 | -0.3171 | 0.2702 | 0.2523 | 0.0939 | 0.1604 | 0.1396 | 0.2512 |
| ENSG00000271503 | CCL5 | -0.5389 | -0.4927 | -0.3872 | 0.3625 | 0.3574 | 0.1157 | 0.1272 | 0.1226 | 0.3334 |
| ENSG00000135114 | OASL | -0.4572 | -0.4593 | -0.3251 | 0.2893 | 0.2886 | 0.0919 | 0.1405 | 0.1531 | 0.2783 |
| ENSG00000187608 | ISG15 | -0.3602 | -0.2821 | -0.2649 | 0.2258 | 0.1939 | 0.1424 | 0.0984 | 0.0530 | 0.1937 |
| ENSG00000089127 | OAS1 | -0.2677 | -0.2440 | -0.2394 | 0.1455 | 0.1517 | 0.0871 | 0.1277 | 0.0793 | 0.1598 |
| ENSG00000112715 | VEGFA | -0.2841 | -0.2620 | -0.4309 | 0.1772 | 0.2255 | 0.1544 | 0.1139 | 0.1963 | 0.1096 |
| ENSG00000117394 | SLC2A1 | -0.2662 | -0.2901 | -0.2396 | 0.1333 | 0.1672 | 0.1164 | 0.1481 | 0.1487 | 0.0823 |
| ENSG00000137628 | DDX60 | -0.2739 | -0.2825 | -0.3385 | 0.1619 | 0.1979 | 0.0716 | 0.1058 | 0.1162 | 0.2414 |
| ENSG00000167767 | KRT80 | -0.2154 | -0.2501 | -0.2249 | 0.0957 | 0.1215 | 0.1405 | 0.1080 | 0.1828 | 0.0418 |
| ENSG00000157601 | MX1 | -0.2235 | -0.2420 | -0.2205 | 0.1191 | 0.1463 | 0.0813 | 0.1140 | 0.0755 | 0.1497 |
| ENSG00000185745 | IFIT1 | -0.2722 | -0.2985 | -0.2534 | 0.1783 | 0.1791 | 0.0846 | 0.1367 | 0.0676 | 0.1778 |
| ENSG00000163644 | PPM1K | -0.2650 | -0.1633 | -0.2712 | 0.0424 | 0.1724 | 0.1089 | 0.1256 | 0.1264 | 0.1238 |
| ENSG00000159399 | HK2 | -0.2544 | -0.2406 | -0.3408 | 0.1248 | 0.1564 | 0.1144 | 0.1587 | 0.2056 | 0.0759 |
| ENSG00000260284 | TPSP2 | -0.2176 | -0.2085 | -0.3111 | 0.0846 | 0.1243 | 0.1526 | 0.1353 | 0.2190 | 0.0215 |
| ENSG00000273184 | AC010655.4 | -0.2052 | -0.1593 | -0.2285 | 0.1392 | 0.0985 | 0.0755 | 0.0999 | 0.1309 | 0.0490 |
| ENSG00000119950 | MXI1 | -0.2541 | -0.2921 | -0.3868 | 0.1735 | 0.1466 | 0.1752 | 0.1300 | 0.1609 | 0.1469 |
| ENSG00000197930 | ERO1A | -0.2247 | -0.1906 | -0.3454 | 0.1161 | 0.1484 | 0.1406 | 0.0899 | 0.1947 | 0.0710 |
| ENSG00000130589 | HELZ2 | -0.2505 | -0.2322 | -0.1873 | 0.1013 | 0.1737 | 0.0533 | 0.1321 | 0.0988 | 0.1108 |
| ENSG00000189060 | H1F0 | -0.2067 | -0.2130 | -0.3058 | 0.1729 | 0.1612 | 0.0845 | 0.0890 | 0.1130 | 0.1046 |
| ENSG00000138642 | HERC6 | -0.1502 | -0.2631 | -0.2216 | 0.1009 | 0.1516 | 0.0311 | 0.1226 | 0.0955 | 0.1333 |
| ENSG00000115548 | KDM3A | -0.2499 | -0.2123 | -0.3794 | 0.1036 | 0.1794 | 0.1374 | 0.1244 | 0.1743 | 0.1226 |
| ENSG00000116260 | QSOX1 | -0.1951 | -0.1752 | -0.2910 | 0.0810 | 0.0946 | 0.1761 | 0.1482 | 0.1580 | 0.0034 |
| ENSG00000075884 | ARHGAP15 | -0.2414 | -0.2604 | -0.2020 | 0.0697 | 0.1705 | 0.0799 | 0.1079 | 0.1642 | 0.1115 |
| ENSG00000173531 | MST1 | -0.2893 | -0.1766 | -0.2288 | 0.1053 | 0.1209 | 0.1786 | 0.1091 | 0.0989 | 0.0818 |
| ENSG00000109046 | WSB1 | -0.2110 | -0.2215 | -0.3464 | 0.0471 | 0.1705 | 0.1484 | 0.1442 | 0.2082 | 0.0605 |
| ENSG00000186868 | MAPT | -0.1906 | -0.1602 | -0.3221 | 0.0959 | 0.1396 | 0.0952 | 0.0639 | 0.1296 | 0.1488 |
| ENSG00000279296 | PRAL | -0.2357 | -0.3126 | -0.2426 | 0.1690 | 0.0896 | 0.2707 | 0.0147 | 0.1507 | 0.0960 |
| ENSG00000125629 | INSIG2 | -0.2486 | -0.1607 | -0.2548 | 0.0986 | 0.0473 | 0.1853 | 0.1813 | 0.1090 | 0.0426 |
| ENSG00000166828 | SCNN1G | -0.2305 | -0.2616 | -0.2847 | 0.0881 | 0.1331 | 0.1105 | 0.1050 | 0.2651 | 0.0750 |
| ENSG00000121858 | TNFSF10 | -0.2322 | -0.1895 | -0.3304 | 0.1334 | 0.1661 | 0.0773 | 0.1304 | 0.1271 | 0.1176 |
| ENSG00000100968 | NFATC4 | -0.1823 | -0.1094 | -0.3804 | 0.1285 | 0.1371 | 0.1400 | 0.0883 | 0.1281 | 0.0500 |
| ENSG00000164096 | C4orf3 | -0.1547 | -0.1956 | -0.3596 | 0.1025 | 0.0980 | 0.2049 | 0.0628 | 0.1801 | 0.0617 |
| ENSG00000138646 | HERC5 | -0.3095 | -0.2617 | -0.2201 | 0.1860 | 0.2265 | 0.0477 | 0.0937 | 0.0929 | 0.1446 |
| ENSG00000250412 | KLHL2P1 | -0.2028 | -0.2012 | -0.1951 | 0.0783 | 0.0480 | 0.1783 | 0.0751 | 0.1362 | 0.0832 |
| ENSG00000014914 | MTMR11 | -0.2667 | -0.1615 | -0.3597 | 0.0582 | 0.1484 | 0.1736 | 0.0811 | 0.1971 | 0.1296 |
| ENSG00000217128 | FNIP1 | -0.2015 | -0.2372 | -0.1978 | 0.0469 | 0.1832 | 0.0181 | 0.1158 | 0.1992 | 0.0733 |
| ENSG00000133321 | RARRES3 | -0.2556 | -0.2654 | -0.2492 | 0.1742 | 0.1222 | 0.1277 | 0.1130 | 0.1193 | 0.1139 |
| ENSG00000172638 | EFEMP2 | -0.3596 | -0.2508 | -0.3300 | 0.0929 | 0.2361 | 0.1334 | 0.1032 | 0.2075 | 0.1674 |
| ENSG00000134326 | CMPK2 | -0.2108 | -0.2662 | -0.2946 | 0.1558 | 0.1126 | 0.0872 | 0.0773 | 0.0679 | 0.2707 |
| ENSG00000105559 | PLEKHA4 | -0.2380 | -0.2708 | -0.4201 | 0.2071 | 0.1809 | 0.1245 | 0.1495 | 0.1464 | 0.1204 |
| ENSG00000185008 | ROBO2 | -0.2168 | -0.2042 | -0.3448 | 0.1180 | 0.1886 | 0.0950 | 0.0496 | 0.1578 | 0.1567 |
| ENSG00000168062 | BATF2 | -0.2068 | -0.2239 | -0.2876 | 0.1702 | 0.1617 | 0.1343 | 0.0502 | 0.0984 | 0.1034 |
| ENSG00000279407 | AC007191.1 | -0.2710 | -0.2153 | -0.2170 | 0.0374 | 0.1800 | 0.0849 | 0.1292 | 0.1734 | 0.0986 |
| ENSG00000105137 | SYDE1 | -0.1463 | -0.2486 | -0.2939 | 0.1422 | 0.0762 | 0.0899 | 0.2337 | 0.1227 | 0.0241 |
| ENSG00000147852 | VLDLR | -0.1761 | -0.2500 | -0.3695 | 0.0855 | 0.0494 | 0.1833 | 0.1900 | 0.1880 | 0.0994 |
| ENSG00000163009 | C2orf48 | -0.2060 | -0.2462 | -0.3168 | 0.0494 | 0.0603 | 0.1609 | 0.1719 | 0.2517 | 0.0748 |
| ENSG00000203985 | LDLRAD1 | -0.2834 | -0.1726 | -0.3028 | 0.0481 | 0.0972 | 0.2139 | 0.1460 | 0.2671 | -0.0134 |
| ENSG00000100342 | APOL1 | -0.1611 | -0.3348 | -0.2233 | 0.0932 | 0.2231 | 0.1176 | 0.0607 | 0.0814 | 0.1431 |
| ENSG00000261649 | GOLGA6L7P | -0.2459 | -0.1906 | -0.3482 | 0.0824 | 0.0631 | 0.2093 | 0.1009 | 0.2778 | 0.0511 |
| ENSG00000186314 | PRELID2 | -0.2497 | -0.2342 | -0.4211 | 0.0617 | 0.1935 | 0.1554 | 0.1969 | 0.1536 | 0.1439 |
| ENSG00000243649 | CFB | -0.2908 | -0.2062 | -0.2000 | 0.1301 | 0.1728 | 0.1124 | 0.0485 | 0.1812 | 0.0520 |
| ENSG00000235706 | DICER1-AS1 | -0.2265 | -0.2680 | -0.1569 | 0.1364 | 0.0365 | 0.1360 | 0.1107 | 0.1669 | 0.0650 |
| ENSG00000117228 | GBP1 | -0.2942 | -0.2694 | -0.1717 | 0.1984 | 0.1959 | 0.0732 | 0.0108 | 0.1374 | 0.1196 |
| ENSG00000182179 | UBA7 | -0.1551 | -0.2395 | -0.4169 | 0.1138 | 0.1188 | 0.1818 | 0.1675 | 0.2153 | 0.0143 |
| ENSG00000204261 | PSMB8-AS1 | -0.1947 | -0.3666 | -0.2562 | 0.1184 | 0.1136 | 0.1929 | 0.1915 | 0.1367 | 0.0642 |
| ENSG00000164849 | GPR146 | -0.4565 | -0.1041 | -0.3634 | 0.1737 | 0.1123 | 0.2024 | 0.1123 | 0.2604 | 0.0631 |
| ENSG00000180777 | ANKRD30B | -0.1799 | -0.1868 | -0.2754 | 0.0812 | 0.2283 | 0.0465 | 0.0556 | 0.1292 | 0.1014 |
| ENSG00000128422 | KRT17 | -0.1851 | -0.2931 | -0.2344 | 0.1269 | 0.1766 | 0.0775 | 0.0639 | 0.1591 | 0.1086 |
| ENSG00000272341 | AL137003.2 | -0.1564 | -0.1799 | -0.2490 | 0.0255 | 0.0857 | 0.1552 | 0.1247 | 0.1643 | 0.0299 |
| ENSG00000167771 | RCOR2 | -0.1755 | -0.1310 | -0.3482 | 0.0869 | 0.0430 | 0.1589 | 0.1677 | 0.1463 | 0.0518 |
| ENSG00000270168 | AC004233.2 | -0.2349 | -0.1637 | -0.4165 | -0.0068 | 0.1231 | 0.1752 | 0.1801 | 0.2572 | 0.0862 |
| ENSG00000240758 | AC010655.2 | -0.3120 | -0.1605 | -0.2921 | 0.1597 | 0.1914 | 0.0612 | 0.1643 | -0.0056 | 0.1935 |
| ENSG00000167550 | RHEBL1 | -0.2806 | -0.1862 | -0.2364 | 0.1903 | 0.1149 | 0.1415 | -0.0002 | 0.1743 | 0.0825 |
| ENSG00000105141 | CASP14 | -0.1317 | -0.1549 | -0.2783 | 0.1022 | 0.0888 | 0.0679 | 0.1152 | 0.1088 | 0.0819 |
| ENSG00000220161 | LINC02076 | -0.2200 | -0.1947 | -0.2405 | -0.0149 | 0.0780 | 0.1979 | 0.1082 | 0.1486 | 0.1373 |
| ENSG00000125895 | TMEM74B | -0.2344 | -0.1177 | -0.4349 | 0.0968 | 0.0820 | 0.1973 | 0.1295 | 0.1736 | 0.1078 |
| ENSG00000148488 | ST8SIA6 | -0.2134 | -0.1765 | -0.3616 | 0.1076 | 0.2025 | 0.0743 | 0.1508 | 0.1326 | 0.0838 |
| ENSG00000064205 | WISP2 | -0.2116 | -0.1711 | -0.2295 | 0.0584 | 0.0819 | 0.1823 | 0.1772 | 0.2143 | -0.1019 |
| ENSG00000119630 | PGF | -0.1908 | -0.1391 | -0.2433 | 0.1407 | 0.0654 | 0.1143 | 0.0623 | 0.2201 | -0.0296 |
| ENSG00000105479 | CCDC114 | -0.1215 | -0.1775 | -0.3418 | 0.0530 | 0.0112 | 0.2254 | 0.1161 | 0.1807 | 0.0545 |
| ENSG00000134321 | RSAD2 | -0.5707 | -0.4688 | -0.5286 | 0.2104 | 0.2307 | 0.8342 | 0.0449 | 0.0449 | 0.2031 |
| ENSG00000109971 | HSPA8 | 0.1766 | 0.1553 | 0.2477 | -0.1519 | -0.0644 | -0.1164 | -0.1326 | -0.1132 | -0.0011 |
| ENSG00000120694 | HSPH1 | 0.1975 | 0.2209 | 0.3261 | -0.2106 | -0.1992 | -0.0787 | -0.0537 | -0.0972 | -0.1051 |
| ENSG00000151835 | SACS | 0.1428 | 0.1412 | 0.0557 | -0.0046 | -0.0720 | -0.0747 | -0.0913 | -0.0613 | -0.0358 |
| ENSG00000260804 | LINC01963 | 0.1677 | 0.1753 | 0.2107 | -0.0422 | -0.0913 | -0.1268 | -0.0623 | -0.1378 | -0.0933 |
| ENSG00000134201 | GSTM5 | 0.1738 | 0.0802 | 0.3169 | -0.0466 | -0.2263 | -0.0521 | -0.1069 | -0.1049 | -0.0341 |
| ENSG00000147642 | SYBU | 0.1547 | 0.0629 | 0.2536 | -0.0681 | -0.0204 | -0.1522 | -0.1522 | 0.0132 | -0.0915 |
| ENSG00000165949 | IFI27 | -0.5273 | -0.4624 | -0.7275 | 0.3474 | 0.2589 | 0.3102 | 0.2561 | 0.2474 | 0.2973 |
| ENSG00000137959 | IFI44L | -0.6802 | -0.5351 | -0.6898 | 0.3414 | 0.3849 | 0.2425 | 0.3254 | 0.2552 | 0.3557 |
| ENSG00000129521 | EGLN3 | -0.2084 | -0.1154 | -0.0724 | 0.1319 | 0.1050 | -0.0370 | 0.1091 | 0.0978 | -0.0105 |
| ENSG00000006459 | KDM7A | -0.1255 | -0.1544 | -0.3141 | -0.0049 | 0.2029 | 0.0781 | 0.0922 | 0.1009 | 0.1248 |
| ENSG00000188177 | ZC3H6 | -0.0487 | -0.0419 | -0.2515 | 0.0604 | 0.0977 | 0.0226 | 0.0182 | 0.0032 | 0.1398 |
| ENSG00000123700 | KCNJ2 | -0.1114 | -0.1114 | -0.1476 | 0.0450 | 0.1105 | 0.0327 | 0.0092 | 0.0589 | 0.1140 |
| ENSG00000259848 | AC097374.1 | -0.2543 | -0.1089 | -0.0567 | -0.0830 | 0.1170 | 0.1255 | 0.1033 | 0.1753 | -0.0183 |
| ENSG00000260710 | AC120498.4 | -0.0661 | -0.3243 | -0.0529 | -0.0577 | 0.0811 | 0.0177 | 0.0606 | 0.2169 | 0.1247 |
| ENSG00000100027 | YPEL1 | -0.1725 | -0.1670 | -0.0279 | 0.0064 | 0.0243 | 0.0549 | 0.0710 | 0.2590 | -0.0482 |
| ENSG00000246859 | STARD4-AS1 | -0.0848 | -0.1023 | -0.2696 | 0.1087 | 0.1289 | 0.1266 | 0.0196 | 0.0329 | 0.0401 |
| ENSG00000196337 | CGB7 | -0.0384 | -0.2025 | -0.2219 | 0.0019 | 0.1765 | -0.0368 | 0.0575 | 0.1510 | 0.1126 |
| ENSG00000058335 | RASGRF1 | -0.0862 | -0.0895 | -0.1026 | -0.0152 | 0.0411 | 0.0909 | 0.0823 | 0.1139 | -0.0347 |
| ENSG00000136514 | RTP4 | -0.2123 | -0.2026 | -0.1315 | 0.0871 | 0.1033 | 0.0193 | 0.0598 | 0.1999 | 0.0770 |
| ENSG00000125954 | CHURC1-FNTB | -0.1632 | -0.3282 | -0.2549 | 0.0082 | 0.0035 | 0.0898 | 0.0937 | 0.1111 | 0.4399 |
| ENSG00000173727 | AP000769.1 | -0.4094 | -0.2255 | -0.5885 | 0.0993 | 0.0750 | 0.4734 | 0.3549 | 0.2589 | -0.0380 |
| ENSG00000104419 | NDRG1 | -0.3641 | -0.3786 | -0.7547 | 0.2366 | 0.3017 | 0.2400 | 0.2776 | 0.3203 | 0.1213 |
| ENSG00000114268 | PFKFB4 | -0.3288 | -0.3261 | -0.6281 | 0.2237 | 0.2224 | 0.2053 | 0.2342 | 0.2720 | 0.1254 |
| ENSG00000151640 | DPYSL4 | -0.3752 | -0.4061 | -0.6555 | 0.1834 | 0.1565 | 0.3988 | 0.2757 | 0.3310 | 0.0915 |
| ENSG00000272870 | AC097534.2 | -0.2239 | -0.3331 | -0.7684 | 0.2639 | 0.1639 | 0.2628 | 0.3531 | 0.1777 | 0.1041 |
| ENSG00000109107 | ALDOC | -0.2270 | -0.2545 | -0.5606 | 0.1123 | 0.1581 | 0.1973 | 0.1873 | 0.2458 | 0.1413 |
| ENSG00000111674 | ENO2 | -0.3117 | -0.2755 | -0.6112 | 0.1417 | 0.1977 | 0.1936 | 0.2207 | 0.3071 | 0.1375 |
| ENSG00000133805 | AMPD3 | 0.1061 | -0.2215 | -0.5263 | 0.0996 | 0.3291 | 0.0453 | 0.1043 | 0.1382 | -0.0750 |
